# Radicalization from a societal perspective

**DOI:** 10.3389/fpsyg.2023.1197282

**Published:** 2023-05-23

**Authors:** Delaram Shafieioun, Hina Haq

**Affiliations:** Institute of Cognitive Science, Osnabrück University, Osnabrück, Germany

**Keywords:** terrorism, radicalization, discrimination, marginalization, social-injustice, power, society

## Abstract

Studies on radicalization tend to focus on the dynamics of extremist groups and how they exploit grievances of vulnerable individuals. It is imperative, however, to also understand the societal factors that lead to such vulnerabilities and grievances. Our social environment plays a key role in how we view the world and shape our beliefs. By understanding the social dynamics, we can gain insight into the motivations that drive people to extremism. Throughout this paper, we examine the societal factors and processes such as discriminative institutional structures and social norms/practices that can make an individual vulnerable and serve as a driving force for them to join a radical group. To do that, we use the process-oriented psychology of Arnold Mindell and the phenomenology of whiteness of Sara Ahmed as our theoretical framework. These frameworks help us map out the societal dynamics causing individuals to carve social niches out of their current social group and into an extremist group. We use interviews with ex-militants of the radical group, Islamic State of Iraq and Syria, to show how certain societal dynamics, such as social injustice, misuse of power, marginalization and discrimination, served as key factors that led these individuals to identify and sympathize with radical ideology. The aim of this paper is to emphasize that, to develop effective preventative measures against recruitment into extremist groups, it is imperative to have a profound understanding of the social dynamics that make an individual susceptible to radicalization in the first place.

## Introduction

As social beings, humans live in societies that are shaped by shared norms, values, and beliefs. Members of society interact with each other and with the world around them to create a sense of identity and purpose. However, there can always be an element of social inequality in society. Dorling (2010) explains that social inequality persists in a society in such a way that it becomes the part of everyday normal life. As a result of social injustice, prejudice and discrimination may become normalized. A pattern of discrimination and stigmatization can result in feelings of rejection, self-stigmatization, internalized shame, and lowered self-esteem (Burke and Parker, 2007), leading to eventual reaction. This reaction can take a variety of forms. As a group, the reaction may take the form of protest, which, if provoked further, may lead to riots. One recent example of this is the Black Lives Matter movement, which was triggered by anger following the murder of George Floyd in Minnesota on May 25, 2020, by a white police officer.

Individuals are also affected by systemic discrimination and marginalization. Marginalization and discrimination may lead to feelings of alienation, exclusion, and deprivation. These feelings can change an individual’s perspective on society. In such a case, she may choose to leave the said society or join an ideology or group that offers her a chance to get her grievances addressed. In recent years, radical groups like Islamic State of Syria and Iraq (ISIS) have also exploited societal injustices to recruit marginalized people. In this paper, we will discuss this affect. This is especially true in the case of people who join extremist groups or extreme ideologies. According to the Council of The European Union (2014) report “Violations of human rights can give rise to grievances and the very conditions conducive to the spread of radicalization and recruitment to terrorism.” Therefore, it is vital to ensure that human rights are respected and protected in order to reduce the risk of extremism.

This paper aims to emphasize that understanding the social dynamics that make individuals susceptible to radicalization in the first place is critical to developing effective preventative measures against recruitment into extremist groups. A review of radicalization studies will be presented in the following section, along with a discussion of why we must take a step back to understand the social imbalance of power in a society in order to understand the reasons that make it inevitable for someone to move toward extremism.

## Extremism and society

Globally, there has been a rise in religious extremism and right-wing extremism. In recent years, European countries have adopted a number of National Action Plans to combat radicalization and violent extremism. A major focus of these action plans is to promote basic and practical research into the root causes and processes that lead to violent engagement (Ajil, 2022). Many of these studies focus on individual and group dynamics without taking into account broader socio-political issues (Sedgwick, 2010; Kundnani, 2012; Ahmad and Monaghan, 2019). This paper aims to fill that gap in the literature by emphasizing the role that societal structures play in radicalization. Radicalization is a multi-pathway, multi-factor complex process (e.g., McCauley and Moskalenko, 2008; Kruglanski and Webber, 2014; Hafez and Mullins, 2015). It is essential that we understand all possible pathways and processes involved in radicalization in order to develop an effective prevention plan. In this paper, we will illustrate how society itself can play a critical role in influencing an individual’s decision to move toward the path of extremism.

In the past decade, there has been an increase in support for and direct participation in radical groups. For example, when the Islamic State of Iraq and Syria (ISIS) movement took hold, almost 40,000 foreigners joined from 130 countries (Barrett, 2017). This raises the question of why people leave their home countries and risk their lives to join radical groups. To understand this, researchers in the field of radicalization studies have identified different factors and processes that pave the path for individuals to adopt a radical ideology. Some of these factors and processes include uncertainty in life (Hogg and Adelman, 2013; Hogg et al., 2013), collective identity problems (Moghaddam, 2012), experiencing alienation (Horgan, 2008; Wilner and Dubouloz, 2010), unsuccessful integration, political grievances which are usually combined with moral outrage and feelings of revenge (e.g., McCauley and Moskalenko, 2008; Sageman, 2008; Schmid, 2013), quest for significance, i.e., the fundamental human need for belonging, respect, performance and self-esteem (Kruglanski et al., 2014), and manipulation of so-called positive emotions like pride, feeling of power, love and the sense of belonging by radical groups to attract and retain recruits (Haq et al., 2020).

The above-mentioned processes and factors are valuable and illuminate various aspects of the complexity of radicalization. But in order to understand the complete picture of radicalization we also need to understand why some people feel a certain way in the first place (e.g., humiliated, alienated, marginalized, etc.), which makes them vulnerable toward radical ideologies. We need to understand the structures in society that construct an environment of mistrust, discrimination, and alienation for certain individuals, and encourage them to seek out groups that promise acceptance and belonging. Our aim in this paper is to emphasize the importance of acquiring a better understanding of the social dynamics that push vulnerable individuals out of society.

The literature on radicalization studies focuses, to some extent, on social aspects that can lead to radical pathways. On the practical level, more emphasis is placed on counter-narratives that challenge predominantly the ideologies of radical groups. For instance, organizations such as the International Center for the Study of Violent Extremism (ICSVE) are focused primarily on combating radical narratives by creating counter narrative videos. As with policies at the government level, the emphasis is more on profiling individuals who may be “vulnerable” to radical ideologies. These profiles target individuals based on their appearance, religion, race, etc. (Blackwood et al., 2015; Abbas, 2017; Schclarek Mulinari, 2019; Abbas et al., 2021). A more blunt statement would be that the governmental efforts to prevent and counter terrorism in the United States and Europe, especially after 9/11, are primarily based on the use of racial profiling. For instance, in the case of Muslim immigrants in Europe who experience Islamophobia on behalf of the host population can induce the feeling of being ashamed or humiliated (Kruglanski and Webber, 2014, p. 381). The feeling of shame and humiliation, combined with systematic discrimination and marginalization based on governmental policies or on lasting prejudices in society can result in creating an environment for the targeted people which make them feel that they are forcefully pushed out of their own society.

In order to enhance our knowledge about the radicalization process and create better counter-radicalization and prevention policies, we have to take a step back, and look at the existing social structures that could act as “push” factors for some individuals or groups to the extent that they leave their society and join radical groups and movements. Some of these “push” factors can be identified as systematic discrimination, racism and marginalization, which are the violation of basic human rights.

The purpose of this article is to provide a deeper understanding of how societal factors such as discrimination, oppression, and racism can facilitate an individual toward the pathway of radicalization. To reach this objective, we rely on Mindell’s (1995) take on the matter of riots and violence in society and the concept of ‘phenomenology of whiteness’ by Ahmed (2007) as our theoretical framework.

In the late 1970s and 1980s, Arnold Mindell developed the approach of process-oriented psychology (also known as process work) in Switzerland. He originally started his work in conflict resolution with individuals and then realized that mere work on an individual level may not be enough to tackle those issues. In his view, although couple therapies, individual therapies, and family therapies are significantly helpful, our constant embeddedness in political, social, or cultural settings demands a framework that considers all of these aspects. As a result, Arnold Mindell started developing other aspects of process work, namely “worldwork” that focuses on working with small and large groups, organizations, and open city forums (Mindell, 2008).

To complement Mindell’s approach on how our constant embeddedness in political, social, and cultural structures is felt and perceived by individuals, we use Ahmed’s concept of “phenomenology of whiteness” (Ahmed, 2007), which accentuates that concepts such as racism are institutional and deeply rooted in our societies. Racism does not belong to history, rather it is ongoing and still lived by some of us, while for others, it is already part of history which is over. Further, we focus on the interviews of some individuals recruited by radical groups, specifically “Islamic State of Iraq and Syria” (ISIS) in order to have a more comprehensive understanding of how these individuals narrate their experience about the “push” factors which they have experienced within their society. Our theoretical framework helps to highlight those less attended aspects of the process of radicalization; namely, how the existing discrimination in societies create a form of social trauma for some individuals and pushes them toward extremism, where they have an opportunity to opt for violence as a solution to their problems.

In the following section, we will start by elaborating on Mindell’s view about issues such as terrorism in society and how they come into existence.

## Societal vs. individual narratives: terrorists or freedom fighters?

When it comes to terrorism-related cases, the media has the power to generate and frame narratives. The impressions we gain from narratives heavily depend on how they are formulated and presented. There is a great deal of misinformation about terrorists in the media and in the legal system because of the way they are portrayed (Mindell, 1995). When an incident happens in the spotlight of the media, it is often the search for an individual motive and the case is interpreted independently from other parallel political movements and social situations. To counter this, Mindell suggests a rather novel definition of terrorism, according to which “[t]errorism is not just a political activity, but a frequent and unseen group interaction based upon the sense of being treated unjustly” (Mindell, 1995, p. 78). He further states that ‘terrorist’ is a word that is used in media discussions to describe people who call themselves ‘freedom fighters.’ This is a unique but important definition since it demonstrates why there are two very different narratives around the same concept. However, portraying terrorism as a “disempowered group’s attack on the mainstream for the sake of equality and freedom” (Mindell, 1995, p. 91) can raise a problem that we would like to clarify before moving on and using that definition. Our connotation with the word ‘terrorism’ is far beyond ‘freedom fighters,’ which implies that these actions are justified and moral. It is important to keep in mind that nothing can justify the act of terrorism and using this definition is not an excuse for justifying terrorism. It is, however, an explanation for why terrorism in some cases happens and how we can understand and counter it more sufficiently. Mindell further elaborates on his definition of terrorism:

Poverty, drugs, joblessness, lack of education, racism, sexism, and social abuse promote violence.^1^ That social injustice foments revenge should be obvious from the fact that the vast majority of those incarcerated for violent acts in all countries come from the groups with the fewest social privileges. In other words, violence occurs, in part, because the oppressed cannot defend themselves from the intentional and covert use of mainstream rank.^2^ (Mindell, 1995, p. 78)

In the case of ISIS formation as a group, the *Hague Center for Strategic Studies* report (Oosterveld et al., 2017) mentioned that to some ISIS appeared out of nowhere and was born suddenly around 2013–2014 when this organization caught international attention owing to its proclamation of a state and broadening its borders. “It is clear that ISIS is a distinct product of its time, geography and circumstances: it grew out of the convulsions of the war in Iraq (2003–2011), the Arab revolutions (2010-present) and the civil war in Syria (2011-present)” (Oosterveld et al., 2017, p. 5). ISIS came into existence as a result of constant war, instability, and extreme poverty in the area. They propagate their ideology in a way that influences people, especially Muslim minorities, in different countries. This is done by taking advantage of the social oppression and marginalization that vulnerable individuals experience in their societies (Speckhard and Ellenberg, 2020). ISIS propagate their ideology in a way that it looks like they are offering vulnerable individuals a chance to stand up against injustice and take revenge for the humiliation and discrimination which they (and other Muslims around the world) are experiencing. The stated mission/agenda of ISIS is to “re-establishing a 7th century Caliphate” and reaffirmation of Islamic norms to guide Arab societies (Oosterveld et al., 2017). In other words, ISIS propagate that they offer the possibility of living a utopian life under the so-called Khilafat they have created.

The interviews by the International Center for the Study of Violent Extremism (ICSVE) show how the above-mentioned factors promote violence as a reaction against felt exclusion and oppression. Especially poverty fosters the feeling of insecurity, as well as lack of education and oppression may breed humiliation and frustration. These examples indicate how oppression in different forms led the individuals to become a part of ISIS.

Abu Ghazwan, a 33-year-old former ISIS member, was imprisoned in Iraq because of a family matter and despite his innocence, the officials did not drop the charges because he was Sunni, and his friends were released because they were Shia. He describes his motivation as follows:

We joined ISIS because we are Sunnis, so that we, Sunnis, become one hand and take over the country, so that it will become a Sunni country, and everyone takes what is his. You take your rights back from those who hurt you. [I believed that Iraq] will become a Sunni country and I will take my revenge on those who imprisoned and hurt me. (Rewards of Joining the Islamic State, 2018)

Salma, a 22-year-old former ISIS member who left Belgium to follow her father to Syria after her father told her: “Life is better here. You can wear your whole hijab. We’re not oppressed here” (A Belgian Family in The Islamic State, 2018). She claims she has not even watched one propaganda video before moving to Syria and her dad’s words were enough for her to leave Belgium.

Another interview featured Albert Berisha, a 29-year-old Kosovar. He left Kosovo to fight in Syria. He mentions the following as his motivation behind his actions:

We saw images of people who were constantly being tortured [by the Syrian regime]. We saw images of killed children. We saw the images of massacred children. We saw inhumane behavior towards women. We saw people being burnt alive. We saw bombings where entire families were killed at once. We saw events as they were unfolding. We had a live stream of the events [on YouTube], so to speak. We experienced almost the same scope and nature of events in the [1999] Kosovo [war] as well. We were also the victims of an unjust regime. But, during the war in Kosovo, I was a child. Then, I couldn’t join the war to fight alongside the Kosovo Liberation Army (KLA). I was only 12 or 13 years old. Now, at [my] age, you can’t just cross your arms and do nothing if you see that the same injustice is happening to someone else. (Islamic State Live Streaming in Kosovo, 2019)

These examples help to shed light on how important it is to de-individualize terrorism. Looking at the overall picture shows that most of the individuals who get radicalized are not subject to a “mental disorder or dysfunction.” Or “should not be pathologized.” They are humans with histories of injuries and experiences of violence without having enough strength or resources to defend themselves or their families. Some of them have the basic goal of “gaining economic support, freedom and the respect necessary to survive” (Mindell, 1995, p. 100).

Mindell (1995) mentions that for minorities and many disenfranchised groups, life has been full of hatred, violence, and revenge. The more invisible and insignificant minorities feel in their environment, the more furious they become. The less the society focuses on the issues that these individuals face, the louder it receives the message: “‘Wake up! You are on a trial! If you do not listen to us, we’ll put a bomb in your home. That should *wake you up*’” (Mindell, 1995, p. 94). Repressed anger can form a desire for revenge. Passivity is a sign of revenge, and it can have different forms. Passivity in forms of shock, shame, numbness, and anxiety can show that someone might have a desire to get revenge and either does not know how or is afraid to do so with the fear of retaliation.

It is important to notice these early signs and listen to people who feel hurt. Listen to their stories and give their anger, shame, and numbness a legitimate platform to be expressed. Otherwise, it can result in violent responses. For instance, Brym (2007) writes that emotions like anger and hate, when combined with opportunities for revenge, act as a motivation for Palestinian bombers into action. Radical groups like ISIS provide this opportunity for vulnerable individuals and shape their affects according to what the group desires. There is a tendency in societies to ignore the early signs of revenge and passivity, and by ignoring these signs, people neglect the problems in the margin and neglect the necessity for change (Mindell, 1995). When the early signs are ignored, revenge alters its forms. It might start with a demonstration against authorities, riots, civil disobedience, and finally turn to a revolution to make the cultural changes that are blocked (Mindell, 1995). Ignoring social marginalization and discrimination by pushing marginalized people away and trying to silence them does not make their voices go away. Instead, they might rather become louder and louder until they are heard. Before moving any deeper, it is important to clarify once again that we are not justifying violence and crimes but trying to understand the anger and frustration which can result in violent riots.

Mindell further elaborates that the political leaders suppress people who are angry in order to keep their popularity and show political success. In the first encounter, this position might seem reasonable, but it totally ignores where anger and violence come from. Why are some people angry? Privileged people, in terms of belonging to the majority and belonging to the center of the society, get angry when their world and its cultural norm is not recreated. For instance, how some conservative and far right governments target minorities. They condemn actions of minorities such as riots and retaliation and suppress them without taking a peek at the roots (Mindell, 1995). As a result, suppression leads to even more revolts, more unhappiness, and does not make conflict and violence disappear (Mindell, 1995).

To understand the anger and frustration of suppressed groups in more detail, in the next section, we will discuss the power imbalance that enables one group to suppress another, resulting in an affective push toward radicalization. To do so, we rely on Mindell’s idea of rank. Which refers to the imbalance of power in a society. The concept of rank helps us to understand that when privileged groups abuse their power, they create an environment that normalizes discrimination and further marginalizes vulnerable groups. This results in anger and frustration among them.

## Social repercussions of marginalization

Mindell argues that issues such as “riots” or “minority crime” are related to their so-called “rank.” He defines rank as “the sum of the person’s privileges” in the society, for instance, color of skin, education, social class, etc. (Mindell, 1995, p. 28). He argues that problems do not necessarily start from the mere existence of ranks, rather they develop when the rank, or in other words the privileges, get forgotten by the ones who have a higher rank. For instance, an educated person might assume that people with less education are ignorant and that they are the ones who cause the problems. On an international level, powerful countries might blame the countries with less power for being violent and supporting terrorism because privileged nations, even if they do the act of international killing in less powerful countries, are always associated with being “victims of terrorism” (Mindell, 1995, p. 90). One recent example is the refusal of the United States of America to let the International Criminal Court (ICC) investigate the cases over the alleged war crimes (involving torture and cruel treatment, dehumanizing abuses, and rape and other forms of sexual violence) committed by U.S. Army and CIA personnel during the invasion of Afghanistan (Scheffer, 2020).

In different societies, people with a specific profile have a higher rank. As a result of having a higher rank, they form the center of that society. People who do not represent the mainstream profile are pushed to the margins. Along this line of thought, Ayata (2019) suggests that the affective dimension of citizenship helps to understand how affects and emotions are used to reinforce differences and differential treatments among the members of a society:

… while two individuals may be equal citizens from a legal point of view, their perceived difference in terms of religion, race, sex, gender, or class may result in identifying one individual as the proper, true citizen who is naturally entitled to the privileges and status of citizenship, whereas the other may be identified as a “quasi” or “technical” citizen, whose belonging to the political community remains in question despite holding citizenship. (Ayata, 2019, p. 332)

When power is abused, not only people are pushed to the margins, but there is also a tendency of marginalizing these people’s problems because what is experienced in the center is deemed to be more important and needs to be focused on. The ones in the center might take an attitude and send an unintended message to the margin: “Stop nagging, quit complaining and fix your problem on your own” (Mindell, 1995). Rejecting people and their problems from the center and accepting that they and their problems belong to the margin is a form of oppression. Mindlessly oppressing and marginalizing individuals has consequences. The ones who are oppressed either become silent or gain vengeance by becoming the oppressors (Mindell, 1995). The overall affective environment created by the ‘ruling class’ or ‘center’ results in clear discrimination, which may lead to fostering frustration in the quasi citizen and result in conflicts. In order to understand how power imbalance in the societal structures affects individuals, in the following section, we would like to use Sara Ahmed’s idea about the ‘phenomenology of whiteness.’ Ahmed’s ideas about the “white world” and its varying impacts on non-white individuals provide a powerful context for Mindell’s work, demonstrating how their perspectives can be further enriched.

## Phenomenology of whiteness

Phenomenology attends, in general, to the tactile, vestibular, kinesthetic, and visual character of embodied reality. However, underlying all of these characters, as Frantz Fanon says, we should consider and think of a “historic-racial” scheme (Fanon, 1986, p. 17). Ahmed (2006) describes our societies as containing a historical and racial dimension. In her view, these dimensions form individuals’ experiences in society differently. As we arrive in this world, we are born into an environment that conveys affective, historical, and racial aspects that affect and direct our way of being in the world. The history of the group or nation to which we belong provides us with our first affective environment. This provides an informative perspective on why, and how, certain groups and individuals may feel oppressed, or socially excluded, from traditional mainstream society (e.g., Abdulrehman, in press).

Ahmed (2007) refers to the idea of whiteness not as a biological characteristic of the body that we are born with. Rather, she defines whiteness as an ongoing history and background of experience which make the lived experiences of humans distinct from one another. This history can enable some humans while disabling the others. This concept of “whiteness” thus refers not only (or even primarily) to the color of skin, it rather denotes an orientation that puts people in different categories and creates different experiences. Therefore, we consider it to be a promising concept for an in-depth exploration of the perceived injustice by minorities. Whiteness is a determining element of rank if we consider whiteness as a feature of “the privileged,” a feature that marginalized populations lack. In this regard, “whiteness” is not only relevant for the Muslims in Europe and the United States, but also Muslims in minority sects in other Muslim countries, e.g., Sunnis in Iraq.

Ahmed argues that, because of the history of colonialism, we live in a white world. The world of whiteness is the world which we inherit. The world which is designed and has orders in a specific way. When we come to this world, we already have a place in which we can dwell, have access, and reach certain objects. This world of whiteness is a world in which certain things are within reach of certain bodies, allowing them to successfully reach those objects while making it difficult for others to do the same. As Ahmed states: “The ‘matter’ of race is very much about embodied reality; seeing oneself or being seen as white or black or mixed does affect what one ‘can do,’ or even where one can go, which can be described in terms of *what is and is not within reach*” (Ahmed, 2006, p. 112).

Ahmed argues that there are some bodies which are seen as “alike”; since they are “sharing whiteness” and have similar objects within reach. By having a common direction, not only does it give the bodies that have the same direction a sense of community, but it also makes them distinct from those with different directions. A *we* emerges as an effect of sharing a common orientation and a *they* emerges as an effect of cohering in a different direction. Ahmed states that the other side of the world is associated with racial otherness, meaning that we attribute all the otherness which we do not recognize as our common characteristics to the other side of the globe (Ahmed, 2006, p. 121). We are we and they are they. The following statement from prime minister of Hungary, Viktor Orbán, is a good illustration of what Ahmed refers to as “racial otherness” and “other side of the world”: “We must state that we do not want to be diverse and do not want to be mixed: we do not want our own color, traditions, and national culture to be mixed with those of others. We do not want this. We do not want that at all. We do not want to be a diverse country” (Bayrakli and Hafez, 2019, p. 42).

In Orban’s statement above, an enormous wish for a distinction between “us” and “them” is visible. The distinction based on the color of skin, traditions and what one calls “national culture” which in *The Fundamental Law of Hungary* is referred to as “Christian Culture” (Bayrakli and Hafez, 2019, p. 48), clearly ignores and devalues the individuals who do not share their mainstream norms.

Ahmed (2006) claims that sharing “otherness” comes at the cost of being stopped. Having a body that is not aligned with “white bodies” can cause two problems. On the one hand, the difficulty of accessing objects because they are far away. On the other hand, the body itself does not cooperate in trailing behind the action. A good example of not having the objects within reach is the concept of ‘glass ceiling.’ The non-white body does not only lack access to certain objects due to living in a white world, but when it attempts to reach for the objects, the non-white body raises against itself and prevents the body from reaching it. The non-white body cannot go unnoticed in the sea of whiteness because the spaces are made in a way that makes the non-white body noticeable. Non-white bodies feel uncomfortable, exposed, and visible as they try to take up space because they do not share a certain likeness with white bodies. Whiteness is the permission for some bodies to pass over repetitively, while the others are being stopped (cf. also Bajwa et al., 2023). Whiteness is invisible to the white bodies because they can fade in the background, whereas the non-white bodies cannot pass and become hyper-visible. Non-white bodies appear to be “out of place,” and therefore being stopped when crossing the line. Not being aware of the invisibility of white bodies to them could be what Mindell referred to as a form of rank abuse and, as already discussed, this could lead to violent responses in the society. Being viewed as an outsider and a constant failure is traumatizing. Being constantly stopped and pushed away can cause a form of social trauma, which could motivate violence from the oppressed group (e.g., Williams et al., 2023).

## Trauma: a facilitator in the radicalization process

In general, a cultural trauma may be described as a physical and psychological assault inflicted by a group that is dominant. This assault is on the culture of a group of people who shares a specific identifying characteristic or affiliation (e.g., ethnicity, religion) (e.g., Stamm et al., 2004; Evans-Campbell, 2008). This trauma can manifest itself in everyday life, often leaving individuals feeling like they are not capable of attaining the same opportunities as those without “otherness.” It can be further compounded when those in positions of power and privilege refuse to acknowledge this injustice, amplifying the feeling of marginalization and exclusion. The “feelings of comfort, unease, anger, empathy, (mis)trust, (dis)respect, love, and hate toward an imagined ‘us’ and ‘others’ are regulated and reproduced in official policies, discourses, and practices” (Ayata, 2019). This systematic discrimination can lead to feelings of deep frustration and resentment, which can lead to further alienation. This plays an important role in the case of young people being recruited to groups like ISIS.

The interviews with former ISIS members and their families conducted by Speckhard and Yayla (2016) indicate a common storyline for the foreign fighters who joined ISIS from Europe, especially Belgium. Many of them were encouraged by teachers from a young age to pursue the (more basic) technical track in school, and then later faced difficulties finding a full-time job. However, even when they have been to the university, they still have to face discrimination in finding jobs and getting hired.^3^ One mother described the situation for her son, a second-generation immigrant, like this: “He was smart and spoke multiple languages, but high school teachers discouraged him and made him feel like he could only be a factory worker or garbage collector, so finally he dropped out of school. Then, of course, he could only get those types of jobs, so he felt totally humiliated. The terrorist recruiter promised him much more” (Speckhard and Yayla, 2016).

In one of the interviews done by ICSVE (2018) which we already referred to, Salma, the 22-year-old Belgian, talks about her experience of joining ISIS. She states: “[In Belgium], sometimes you feel targeted. You feel watched upon as if you are not the same like them. If your head is covered, you are wearing a hijab this big and everything, you are watched upon.” (A Belgian Family in The Islamic State, 2018). Another interview (Georges the Belgian Jihadist, 2018) with Georges M., a 25-year-old from Belgium, who intended to join the uprising in Syria but never succeeded, portrayed the common story of being stopped, becoming hyper-visible and not being able to move upward. He converted to Islam when he was in high school and faced his parents’ disagreement and disappointment. He was suspended from high school for proselytizing, and he ultimately dropped out of the school. “I knew that if I stayed in that establishment or another, things would get worse” (Georges the Belgian Jihadist, 2018). He began working in jobs below his intellect and, because of the lack of high school final certificates, he could not get a university education. He explains that after watching videos from Syria, he and his friends felt the urge to go to Syria and help Muslims fight against Bashar al-Assad. They had to return home because the father of a friend took them back. Here is a snippet demonstrating his perceived the discrimination:

I wish everyone could practice his religion as he wants to like it’s been done for a long time. Not only for Muslims, but also for Christians and Jews. [But, here in Belgium] I cannot pray at work like I want. If I wear a beard, there are prejudices. If my wife wants to wear the hijab, she’ll face discrimination. [At my job], I asked to do my prayers. Everybody goes out to smoke cigarettes. Why can’t I go out to do my prayer that doesn’t take more than five minutes? I am not in an Islamic land, but in a so-called ‘democratic’ country. [Here] there is no trust in the other. (Georges the Belgian Jihadist, 2018)

When societies and institutions fail to provide spaces for some individuals and groups to act freely as compared to the other groups of society, trauma is generated. After 2001, identities of Muslim citizens in America were put under surveillance and many were perceived as dangerous or threatening. This created a comparable affective register that highlights the two categories of citizens, one (who were not Muslims) automatically shift to the naturally entitled citizens, and others (Muslims) whose citizenship became conditional and relegated to a formality if they do not act, feel and behave in a desired way. This required additional emotional and affective efforts to confirm the rightful political belonging (Slaby and von Scheve, 2019). Having the wrong name, the wrong color of skin, the wrong nationality, the wrong religion, obstruct the path for individuals, sometimes temporarily and sometimes forever. It ceases them and their movements. Even if they have the right passport with a wrong body, their way is blocked. Therefore, if our nationality does not match our body, if my name does not match my nationality, if my nationality does not match my religion, then we are held as suspect and should answer those inconsistencies and mismatches. Some bodies feel more at home, and some feel the discomfort of being strangers. Some bodies are recognized more as “strangers” and “out of the place” than others (Ahmed, 2006). Being a stranger is being suspected of sharing otherness.

What non-white bodies are facing is a form of social traumatization, since it targets the entire group, and it is implemented in a societal context. Another factor that aggravates this socially embedded trauma is that the public fails to acknowledge or even actively denies the trauma. A famous example is the debate about Armenian Genocide. Even in trauma-related literature, this avoidance of acknowledging oppressive traumatic experiences as trauma exists. Holmes et al. (2016) criticized DSM 5 for not including different forms of oppression (e.g., racism or sexism) as potentially traumatic events. They elaborate that empirical evidence has shown that marginalized groups experienced higher PTSD levels in comparison to the majority (see Holmes et al., 2016). Despite established empirical evidence, the current definition of trauma fails to include institutional, systemic and psychological forms of violence as potentially traumatic experiences. This kind of psychological violence which is usually “invisible” is a form of social neglect of trauma. As mentioned before, social trauma is an imperative element in the recruitment of young people by groups like ISIS. On the one hand, not being able to move forward, feeling hyper-visible, being stopped and interrogated, and being considered as an outsider and on the other hand, the dream that ISIS sells, the dream of having a home where you can be free and belong to. This can create a powerful contrast between the feelings of hopelessness and the promise of security and belonging that ISIS offers, making the group particularly attractive to vulnerable young people.

When hierarchies and the differences in rank are institutionalized, people with higher rank usually feel that they do not have to bear with the problems of people with less rank. Hence, all the problems get associated with the rank and the rank system gets internalized (Mindell, 1995). Internalization of oppression is so strong that people from minorities feel traumatized. There is no doubt that the political process in each country is different, but there are similar elements in all of these processes: the structure of processes between center and margin. Considering that almost all of our sources are linked to cultures practiced in the center of the society and our embeddedness in cultural systems, how we feel, and think is also an effect of that culture. Subsequently, our sense of self-worth and the worth of others is linked to what we receive from that culture. As a result, it is understandable that marginalized groups may lack confidence. “Unfulfilled needs,” “repressed feelings,” and “the search for the meaning of life” of marginalized groups, play a crucial role in forming a mass frustration (Mindell, 1995, p. 24).

Sometimes people do support social order and let it continue as it is; as if by nature, some are superior to others. For instance, when people see that a considerable proportion of immigrants is unemployed, they start doubting immigrants’ abilities and intelligence instead of asking what would have happened if they would have gotten the same amount of opportunities to unfold their talent as the mainstream population (Mindell, 1995). This discriminatory approach persists even in research literature. Jason Richwine, for example, received a doctorate in public policy from Harvard in 2009. In his dissertation titled “IQ and Immigration Policy,” he argues that immigrants have lower IQs than native white Americans, and that these low IQs are likely to persist for generations to come (Richwine, 2009). In addition, the book “The Bell Curve” by political scientists Charles Murray and Richard Hermsteirrayn can be cited as an example. They argued in the book that poor people, especially poor black people, were intrinsically less intelligent than white people (Herrnstein and Murray, 1996). Mindell (1995) mentions that mass frustration of minorities can ignite a revolution. In other terms, they aim at the replacement of existing structures, i.e., a revolution that replaces the current social, economic, or political structures. Revolutions are more radical than reforms. Reforms are some alternations in the existing systems, whereas revolutions aim to change the entire system. If structures do not change enough with reform, revolution follows (Mindell, 1995, p. 225). The up-rise of ISIS as a group might be a case in point. “It is no coincidence that ISIS and its extreme jihadi message took root in a region that was experiencing socio-political upheavals arguably of a ‘one in a century’ kind” (Oosterveld et al., 2017, p. 9). After the attacks of 9/11, the American government started the war in Afghanistan and Iraq against Al-Qaeda. In the time of Iraq’s invasion, Al-Qaeda was not yet grounded in Iraq, but following years of chaos, the circumstances became ideal for them to expand in Iraq and beyond. There are two concrete fatal decisions on behalf of Coalition Provisional Authority (CPA), which assisted the rise of ISIS, namely the de-Baathification of Iraq’s government and disbanding the Iraqi army. These decisions played an important role in promoting and increasing Iraq’s sectarian conflicts that played off Sunni against Shia. Since the CPA’s decision had an exclusive impact on Sunni population, Al-Qaeda which later became the Islamic State of Iraq (ISI), found an opportunity and used the massive frustration of the Sunni population against the Shia and Western forces (Oosterveld et al., 2017). Not to mince words, the mass frustration of the Sunni population with their governments and Western countries for the marginalization, the help of former Iraqi Baathist officials as a significant part of ISIS’ leadership, high goals of living in a society without discrimination under Islamic laws, the failure of the governing powers in Syria and Iraq, and lastly the unintentional support from the outside by Western countries and rebels in Syria and Iraq, contributed to the rise and development of ISIS.

In the following section, we highlight how the increase in Islamophobia might have an imperative role in contributing to a heightened sense of paranoia and fear of Muslims, adding to the appeal for the vulnerable individuals to join the extremist groups like ISIS.

## The double signal of islamophobia

According to Mindell (1995), being unaware of one’s rank could be a trigger for conflicts in the society. He elevates his argument and introduces the concept of “sending mixed signals” as one of the most troublesome consequences of unawareness of rank. In our communication, within families, groups, communities, and even on an international level, two types of signals are sent. “Primary signals” are the ones that are intended, and “secondary signals” are the signals which are unintended and indicate another level of a person’s feeling and unconscious sense of power and rank. Often we send the primary message of “Let us talk” accompanied by a secondary message of “I am superior and what I say does not come in debate” or “stay where you belong” (Mindell, 1995, pp. 49–60). Despite the primary signal about “respect for religious diversity” the increase of Islamophobia not just among the people, but also among politicians is a secondary signal that is contrasting with the primarily intended message and thereby stigmatizes Muslims in general. As an example of the contrary signals, Canada can be cited. While Canada emphasizes multiculturalism as a primary message, it also conveys a secondary message through institutional policies such as Bills 21 and 62. Policies like these target Muslim women and marginalize them (Williams et al., 2022). In this section, we explain how the arising issue of Islamophobia, prejudice against Muslims and the policies to combat these issues could send such secondary signals.

There has been an increase in Islamophobia and prejudice against Muslims in recent years. Muslim identity has been portrayed as an incompatible identity with modernity and democracy (Wike and Grim, 2010). Muslims, and especially second-generation immigrant Muslims, can have a strenuous life in Western societies. There is unfortunately little research on the matter of “Western views towards Muslims” but research by Wike and Grim (2010) has shown that Western societies perceive Muslims as both a security and a cultural threat which causes negative attitudes toward Muslims and therefore people tend to be more intolerant toward them. Wike and Grim cite from Cesari (2004) that September 11 was a starting point for a “Bin Laden effect” which caused discrimination and even violence against Muslims in Western societies. “The ‘Bin Laden Effect’, according to Cesari, ‘consists mainly of casting all Muslims within the U.S. and Europe in the role of The Enemy, transforming them into scapegoats for the entire society’” (Cesari, 2004). Therefore, the perceived threat identified with Muslims turns the majority (non-Muslims) against the minority. The results of this research have shown that in the investigated countries (Spain, the U.S., Britain, France, and Germany) Muslims are regarded not only as a cultural but also as a security threat. Negative attitudes regarding Muslims do not primarily arise because of Westerners being worried about the incompatibility of Islam with democracy and modernity, rather they lie in the perception of extremism within Muslim communities. Certainly, there are extremist groups both in Muslim countries and in the West. Research has continuously shown that in most Muslim communities, only a small minority holds extremist views, and the majority are against extremism (Wike and Grim, 2010). For instance, in 2015 Pew Research Centre collected data about how the countries with significant Muslim population hold negative views about extremist organizations like ISIS (Poushter, 2015). Perceiving Muslims as a security threat can lead to extreme reactions against them and a sense of exclusion from society.

The European Islamophobia Report 2018 (Bayrakli and Hafez, 2019) was published with the financial support of the European Union on the matter of Islamophobia in Europe. This report was an investigation on the dynamics that support anti-Muslim racism in Europe in a direct or indirect way. A simple example would be the incidents in which Muslims are the target. These are usually described as hate crime, whereas in other cases, they would be referred to as terrorist attacks (e.g., Corbin, 2017). In the following paragraphs, we will provide an overview of this report.

During the last decades, far-right movements, nationalists, and populists started rising in Europe, in countries such as Italy and Austria they even have been in power and in coalitions for a while. Considering that only 12% of Muslims who have been experiencing discrimination report to the authorities (Bayrakli and Hafez, 2019), Islamophobia incidents happen too frequently to be ignored. Austria reported 540 cases of Islamophobic incidents in 2018, which shows a 74% increase in comparison to 2017 in anti-Muslim racist attacks. In Belgium, 84% of reported discriminations at workplaces were related to Islamophobia. France documented a 52% rise of Islamophobic incidents in 2018 in comparison to 2017, with a total number of 676 incidents (which include 20 physical attacks). According to the police statistics in Germany, there were 678 attacks on German Muslims; 40 attacks on mosques, and 1775 attacks on refugees in 2018. In the Netherlands, 91% of a total of 151 incidents of religious discrimination reported to the police were related to Muslims. Violent acts against Muslims happened in different forms, for instance, rape, shootings, planning to commit terrorist attacks against Muslims such as poisoning halal foods, killing imams, physical attacks against Muslim women, and so on.

Another dynamic against Muslims is the use of Islamophobic language by high-ranking politicians. Most of these politicians belonged in the far-right and their Islamophobic language normalizes and decreases the threshold of what is appropriate to be said in public discourses. Using such a language normalizes and legitimizes discrimination of Muslims in the society as citizens. Examples of the use of Islamophobic language by high-rank politicians were collected and reported by the European Islamophobia Report 2018 (Bayrakli and Hafez, 2019): In Belgium, Bart de Wever, NVA leader stated: “Jews avoid conflict that is not the case with Muslims.” In Bulgaria, the Prosecutor Nedyalka Popova mentioned: “At present, according to statistics, Muslims are 10–12% in Bulgaria, and we have no reason to think that they will become less. When they reach 30%, the state is already in danger. They are a monolithic mass, who are easy to manipulate during the elections, and they are almost like a militarized structure. If they have been told to go and vote, they go.” In the Czech Republic, Dominik Hanko, vice-chair of the SPD party in the Ústecký district, referred to the Muslim population as “locusts” that destroy everything around them. In Denmark Erik Høgh-Sørensen, a regional council member in Nordjylland and parliamentary candidate for the Danish People’s Party addressed the rejected asylum seekers and said that at Lindholm (detention center for rejected asylum seekers) pork should always be included in all meals of the menu. In Germany, after the Chemnitz incident, the former German Minister of the Interior, Horst Seehofer (CSU), said: “Migration is the mother of all problems.” In Ireland, “the Identity Ireland leader Peter O’Loughlin claimed that Islam was ‘destroying’ cities in Europe and warned of the risk of ‘Sharia courts,’ ‘rape gangs,’ and ‘grooming gangs’ should a mosque be built in Kilkenny. In Italy, the former Minister of Interior Matteo Salvini warned of the danger of Islam in Italy and stated that his future government put an end to the “irregular Islamic presence” in Italy. Geert Wilders, a Dutch politician, produced and spread a campaign video accompanied by horror music and the following text with red letters: “Islam stands for hate against Jews, Christians, women, and homosexuals.” This video ends with the sentence “Islam is deadly” using red drops as a resemblance to blood. In Norway, Per-Willy Amundsen, MP for the Progress Party and former minister of justice, mentioned his right to say that “the migration from Muslim countries should stop.” In Serbia, president Vučić referred to Milošević, who was charged with Muslim genocide, as a great Serbian leader, with good intentions yet bad results. Also, Prime Minister Ana Brnabić stated on the Srebrenica genocide: “[It] was a terrible, terrible crime but… genocide is when you are killing the entire population, the women, children and this was not that case.” Downplaying and denying the genocide could be one example of public failing to acknowledge the trauma. In the UK, Boris Johnson referred to women who wear Burqa as letter boxes and said that is a ridiculous choice to walk around like that (Bayrakli and Hafez, 2019, pp. 40–44).

Unfortunately, the issue of Islamophobia is not limited to Islamophobic language. In some cases, there are enforcements or demands for laws from government or political parties that directly target Muslims and put different restrictions on them in comparison to other religious communities. The European Islamophobia Report 2018 (Bayrakli and Hafez, 2019) provides some examples of this legislation. In Denmark, the Danish government introduced stricter legislation for “Ghetto Package” who are low-income Muslim enclaves, to regulate life in their community. Based on these sets of laws, they receive greater penalties for crime, receive less money from the public section, and have certain restrictions regarding the upbringing of their children. In general, there are 22 rules which the government believes should be applied to achieve their goals. The new set of laws affects not only these special groups of Muslims in Denmark. There has been a reform of the law on daycare, based on which Muslim parents are deprived of the right to choose where they want their children to go to daycare. Another approved law in Denmark is the obligatory handshakes with the local mayor at a citizenship ceremony (Bayrakli and Hafez, 2019). This is potentially a problematic issue for Muslims since physical contact with the opposite sex (with the exception of the family) such as handshaking is discouraged and for some even prohibited.

The more these kinds of laws and open discrimination are enforced, the more marginalized Muslim community gets within the society. These kinds of discriminatory conditions may push the vulnerable individuals toward the extremist groups. The narratives that groups like ISIS use to recruit individuals, highlights the injustice, collective grievances, and discriminations, and also offer a way to fight such injustices. Grievances and its associated emotions can lead individuals or groups of people to search for a platform where they can redress it. The ‘seeking’ phase, as a result of grievance, becomes a ‘vulnerability’ toward radicalization, since it is in such vulnerable situations that individuals are receptive to other worldviews that promise justice and revenge. Radical organizations use their power, resources, and creativity to turn individual grievances and emotions into collective claims and to stage opportunities to act upon these claims (van Stekelenburg, 2017).

If we want to fight extremism, it is not enough to fight how radical individuals deal with injustice. We have to take a step back and also fight the injustice itself. We would like to draw the attention once again on how oppressing the whole group and systematic discrimination can cause the outburst of anger and lead to extremism or as a young second-generation Moroccan man stated: “If all the white Belgians think I’m a monster, then I might as well be one” (Speckhard and Yayla, 2016).

There are not always written laws that are oppressive. There are numerous things that are perpetrated by systemic racism that are not written laws, and it is difficult to prove that they exist and fight against them because on paper, “they do not exist.”

## Conclusion

In our opinion, to tackle the issue of radicalization entails tackling racism and oppression as well. The way we deal with the radicalization in our societies often goes in the direction of pathologizing radical members without considering their situation and backgrounds, before going through the radicalization process. Often the focus is so much on proving the ideology wrong. There is a tendency to forget that these people have many psychological vulnerabilities, often stemming from the discrimination that they experienced in their society. Therefore, it is crucial to address systematic discrimination, racism, institutional abuse, and imbalance of power between different groups in a community to show how these issues can traumatize minorities and how minorities react to this trauma. Groups like ISIS take advantage of this trauma to sell their propaganda and recruit individuals by promising them a life in Utopia and a chance to take revenge on their oppressors. Mindell (1995) believes that the problem of terrorism will not be solved if we just take action on the international level. We have to be ready to deal with the roots steaming within families, churches, mosques, local organizations, and governments. The mainstream finds it difficult to accept that it shares a responsibility in pushing marginalized people toward extremism. People do not show fundamentalist and abusive behavior out of the blue, they have often been badly hurt (Mindell, 1995).

In our view, factors like systematic discrimination, abuse of power, and constant marginalization play a pivotal role in pushing discriminated individuals out of society (in some cases, as illustrated in the examples) and into radical ideologies promising better lives. However, having said that, we do not want to justify and bring excuses for violent actions. We want to stress that these types of violent actions happen in a social context and if we continue treating terrorism just as an indication of inner and individual problems rather than social injustice, we can never fully succeed in solving the issue.

## Data availability statement

The original contributions presented in the study are included in the article/Supplementary material, further inquiries can be directed to the corresponding author.

## Ethics statement

Ethical review and approval was not required for the study on human participants in accordance with the local legislation and institutional requirements. Written informed consent from the patients/participants or patients/participants’ legal guardian/next of kin was not required to participate in this study in accordance with the national legislation and the institutional requirements.

## Author contributions

All authors listed have made a substantial, direct, and intellectual contribution to the work and approved it for publication.

## Funding

This work was supported by the Deutsche Forschungsgemeinschaft (DFG, German Research Foundation)—project number GRK 2185/2 (DFG Research Training Group Situated Cognition).

## Conflict of interest

The authors declare that the research was conducted in the absence of any commercial or financial relationships that could be construed as a potential conflict of interest.

## Publisher’s note

All claims expressed in this article are solely those of the authors and do not necessarily represent those of their affiliated organizations, or those of the publisher, the editors and the reviewers. Any product that may be evaluated in this article, or claim that may be made by its manufacturer, is not guaranteed or endorsed by the publisher.

## Footnotes

^1^Recent research incorporating machine learning also shows that low social status and problematic social relationships can contribute to extremism (Ivaskevics and Haller, 2022).

^2^Mindell defines rank as “the sum of the person’s privileges” in the society, for instance, color of skin, education, social class, etc. (Mindell, 1995, p. 28).

^3^Papers that elaborate on immigrant workers’ discrimination on the European job market, (see Weichselbaumer, 2017, 2020; Ball et al., 2022).
